# Prevalence, awareness, treatment, control of type 2 diabetes mellitus and risk factors in Chinese rural population: the RuralDiab study

**DOI:** 10.1038/srep31426

**Published:** 2016-08-11

**Authors:** Xiaotian Liu, Yuqian Li, Linlin Li, Luning Zhang, Yongcheng Ren, Hao Zhou, Lingling Cui, Zhenxing Mao, Dongsheng Hu, Chongjian Wang

**Affiliations:** 1 Department of Epidemiology and Biostatistics, College of Public Health, Zhengzhou University, Zhengzhou, Henan, PR China; 2 Department of Clinical Pharmacology, School of Pharmaceutical Science, Zhengzhou University, Zhengzhou, Henan, PR China; 3 Department of Nutrition and Food Hygiene, College of Public Health, Zhengzhou University, Zhengzhou, Henan, PR China; 4 Department of Prevention Medicine, Shenzhen University School of Medicine, Shenzhen, PR China

## Abstract

The study aimed to investigate prevalence, awareness, treatment and control of type 2 diabetes mellitus (T2DM), and to explore potential risk factors in rural areas of China. A total of 16413 individuals aged 18–74 years in rural districts were recruited from the Rural Diabetes, Obesity and Lifestyle (RuralDiab) study for the epidemiological research. Meanwhile, a meta-analysis including 7 published studies was conducted to validate the result of the cross-sectional study. The rates of crude and age-standardized prevalence, awareness, treatment and control of T2DM were 12.19%, 67.00%, 62.35%, 22.20% and 6.98%, 60.11%, 54.85%, 18.77%, respectively. The prevalence, awareness, treatment and control of T2DM displayed increased trends with age (*P*_*trend*_ < 0.01) and were strongly associated with education, drinking, more vegetable and fruit intake, physical activity, family history of diabetes, body mass index (BMI). The results of this meta-analysis showed that the pooled prevalence, awareness, treatment and control of T2DM in China countryside were 7.3% (5.3–9.4%), 57.3% (36.9–77.6%), 48.4% (32.4–64.5%) and 21.0% (9.9–32.1%), respectively. The prevalence of T2DM was high with inadequate awareness, treatment and control of T2DM in China rural areas. Healthy lifestyles should be advocated to reduce prevalence and improve awareness, treatment, and control of T2DM in Chinese rural residents.

As the third leading cause of mortality, diabetes seriously threatens to human health worldwide^1^,^2^, and it has caused large disease burden to the patients, their families and the society, especially in developing countries^3^,^4^,^5^. There are many complications of diabetes such as diabetic eye disease and diabetic nephropathy, which could lead to blindness and kidney failure^5^,^6^,^7^. In addition, diabetes increases the risk of cardiovascular diseases^8^,^9^. International Diabetes Federation (IDF) Diabetes Atlas Seventh Edition estimated that there were 415 million adults aged 20–70 living with diabetes and 5.0 million deaths were attributed to diabetes globally in 2015^3^. Furthermore, diabetes causes huge financial burden. In 2015, 673 billion dollars were spent on health expenditure of diabetes alone, which accounted for 12% of total expenditure^3^. However, the prevalence of type 2 diabetes mellitus (T2DM) in almost all countries has increased rapidly in recent decades, especially in developing countries^10^,^11^,^12^,^13^. It was reported that about three quarters of people with diabetes were from low- and middle-income countries^3^. Epidemiological studies have confirmed that T2DM prevention and control were crucial to postpone or decrease its incidence related complications and its financial burden^14^. Nevertheless, the awareness, treatment and control of T2DM were disproportionately low^10^,^13^,^15^. Almost half of people with this disease were undiagnosed all over the world, and the corresponding proportion was 69.83% in Chinese adults^3^,^15^.

The prevalence of diabetes has increased from less than 1.0% in 1980 to 11.6% in 2010 in the Chinese population^15^, which means that more than 100 million of people were suffering from diabetes and China has become one of the largest T2DM population countries in the world. Although the prevalence is higher in cities than that in the countryside, the increase of incidence rate is faster while the awareness, treatment and control of diabetes are lower in rural districts than those in cities^16^,^17^. Therefore, it is necessary to assess the epidemiological characteristics and risk factors of T2DM to take effective interventions in a specific population.

Although previous studies have focused on prevalence, awareness, treatment, control and risk factors assessment in China^17^,^18^, the results were inconsistent due to the difference in living regions, economy, culture, occupation, diet and life style. What’s more, the research special in synthesizing the prevalence, awareness, treatment and control of T2DM by combining rural study and meta-analysis has not yet been reported. Moreover, the meta-analysis only selected articles conducted in the Chinese countryside containing prevalence, awareness, treatment and control of T2DM. In addition, a comprehensive evaluation of T2DM epidemiology nationwide in China rural districts would potentially benefit the prevention and control of diabetes and policy-making. Therefore, the present study synthesizing prevalence, awareness, treatment and control of T2DM was conducted by combining epidemiological research and meta-analysis in China countryside areas.

## Results

### Demographic characteristics

Table 1 shows the demographic characteristics of the participants. Of the 16413 participants, a total of 2000 were diagnosed with T2DM. The crude and age-standardized prevalence of T2DM were 12.19% and 6.98%, respectively. Compared with those without T2DM, subjects with the disease had the following characteristics: older age, higher body mass index (BMI), higher percentage of worse marital status, lower education level, alcohol drinking, physical inactivity and family history of diabetes, lower ratios of smoking, high fat diet and more vegetable and fruit intake (*P* < 0.05 for each).

### Prevalence, awareness, treatment and control of T2DM

Table 2 presents comparison of prevalence, awareness, treatment and control of T2DM between various factors. Among subjects with T2DM, 1340 people were aware of their condition, 1247 taking medicine, and only 444 had their fasting plasma glucose (FPG) level controlled. The crude and age-standardized awareness, treatment and control of T2DM were 67.00%, 62.35%, 22.20% and 60.11%, 54.85%, 18.77%, respectively. There were increasing trends in prevalence, awareness, treatment and control of T2DM with age (*P*_*trend*_ < 0.05). The prevalence displayed a decreased trend as the level of physical activity increased but an increased tendency with higher BMI. The awareness and treatment of T2DM presented tendencies toward decreasing with increasing physical activity and BMI (Table 2).

### Change tendency of T2DM different subgroups

**Figure 1:**
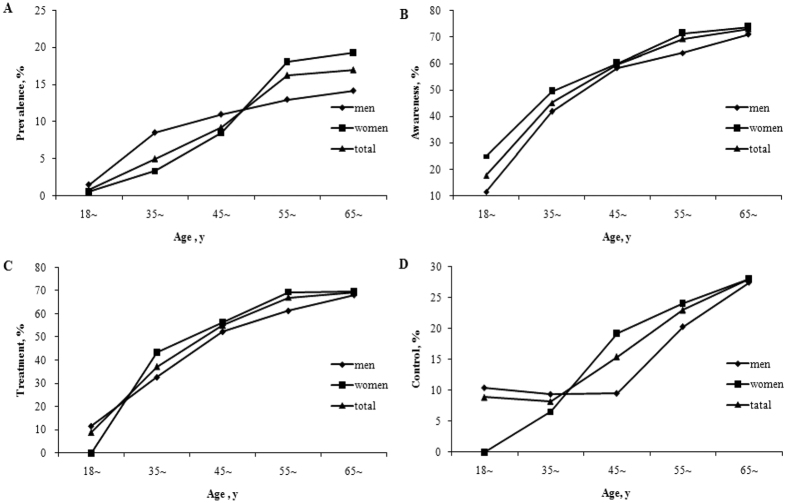
The age-standardized prevalence (**A**), awareness (**B**), treatment (**C**) and control (**D**) of T2DM between different age groups and sex. Figure 1A is for the age-standardized prevalence of T2DM between different age groups for men and women, Fig. 1B for the age-standardized awareness of T2DM, Fig. 1C for the age-standardized treatment of T2DM, and Fig. 1D is for the age-standardized control of T2DM.

Figure 1 shows the inclination of different gender age-standardized prevalence, awareness, treatment and control of T2DM with age. The age-standardized prevalence, awareness, treatment and control of T2DM tended to increase with ageing for both genders while men had higher prevalence of T2DM than women aged younger than 55 years old, but lower at older age (Fig. 1A). The rates of awareness, treatment, and control of T2DM were all higher in women than those in men (showed in Fig. 1B–D).

### Analysis of risk factors

Table 3 describes the results of multivariate logistic regression for prevalence, awareness, treatment and control of T2DM. Compared to younger individuals, older individuals have higher prevalence, greater probability of being aware of their diabetic conditions and seeking medical treatment. Family history of diabetes was significantly related to prevalence, awareness and treatment. High risk drinking, overweight and obese were significantly positively associated with prevalence while education, more vegetable and fruit intake and higher physical activity were negatively associated with prevalence. Never drinking and high physical activity were correlated to lower levels of awareness and treatment of T2DM. No risk factors were significantly related to control of T2DM.

### Meta-analysis

**Figure 2:**
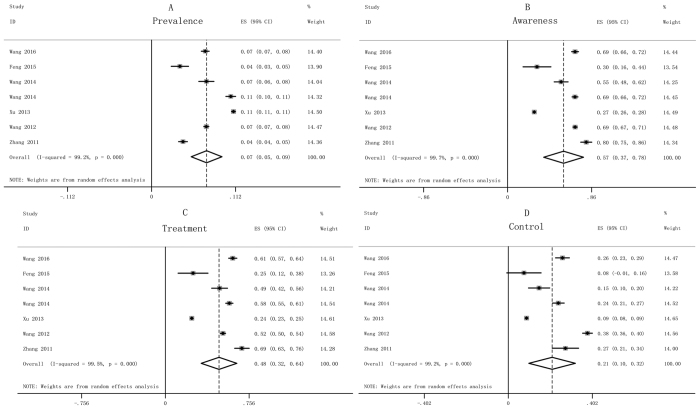
Forest plots of prevalence (**A**), awareness (**B**), treatment (**C**) and control (**D**) of diabetes of all selected studies. The pooled prevalence of T2DM in Fig. 2A, the summarized awareness of T2DM in the Fig. 2B, the pooled treatment of T2DM in Fig. 2C, and the summarized control of T2DM in Fig. 2D.

According to inclusion and exclusion criteria, 7 studies^15^,^19^,^20^,^21^,^22^,^23^,^24^ were selected and a total of 109718 subjects containing 10260 case of diabetes were eligible for the meta-analysis (Supplementary Figure S1 and Supplementary Table S1). The pooled prevalence, awareness, treatment and control of diabetes were 7.3% (5.3–9.4%), 57.3% (36.9–77.6%), 48.4% (32.4–64.5%) and 21.0% (9.9–32.1%), respectively (Fig. 2). There was obvious heterogeneity (*I*^*2*^ > 50%), and publication bias was not found by use of the Begg’s and Egger’s tests (*P* > 0.05).

## Discussion

To our knowledge, the present study is the first one attempting to overall appraise the prevalence, awareness, treatment and control of T2DM in Chinese rural population using the large sample epidemiological research and meta-analysis. The results of this epidemiological study indicated that the crude prevalence, awareness, treatment and control of T2DM were 12.19%, 67.00%, 62.35% and 22.20%, respectively, which were slightly higher than the results of this meta-analysis. In addition, the age-standardized prevalence, awareness, treatment and control of T2DM displayed increased trends with age and the rates were higher in women than those in men.

The ascending trend of prevalence by age in both genders indicated incidence of T2DM in women was higher than that in men at around 50 years old, which might be because women are in the stage of menopausal transition around 50 years old. There were some differences in prevalence (12.19% *vs*. 7.3%), awareness (67.00% *vs*. 57.3%), treatment (62.35% *vs*. 48.4%), and control (22.20% *vs*. 21.0%) between both studies (epidemiological research and meta-analysis). There might be some potential reasons for the phenomenon, such as age structure and economic level of participants. The mean age of participants was 54.58 ± 11.32 years in the RuralDiab study including more older subjects (40.05% of participants aged ≥60 years old). In addition, the samples of the RuralDiab study were from four counties with higher economic level and living standard. The above reasons might have partly contributed to the higher awareness, treatment and control of T2DM in the epidemiology study^3^,^15^,^16^. The further analysis showed that the age-standardized awareness (60.11%), treatment (54.85%) and control (18.77%) rates in present study were unsatisfactory compared with those in developed countries^11^,^25^. Therefore, effective intervention measures should be taken to reduce the prevalence and to improve awareness, treatment and control of T2DM in Chinese rural residents.

A number of studies have shown that age, poor education, family history of diabetes, physical inactivity and overweight or obese were risk factors of T2DM^26^,^27^. Our study findings were in line with the reported researches. Generally speaking, enhancing education level, moderate physical activity and well controlling body weight could reduce the risk of T2DM.

The relationship between alcohol consumption and T2DM remains controversial. A study from Korean demonstrated that high-risk alcohol drinking increased the risk of T2DM in men, but the association was not observed in women^28^. Our study found that only high-risk alcohol drinking was positively related to T2DM. Light-to-moderate alcohol drinking was confirmed to be associated with a lower risk of T2DM^29^. Therefore, there may be a dose-response relationship between alcohol consumption and T2DM.

High fat diet and smoking are well known to be closely associated with T2DM^30^,^31^. However, these associations were not found in our study. Some subjects might have changed their lifestyle after being diagnosed with T2DM, which might have influenced our results^27^. More vegetable and fruit intake is beneficial for the prevention of T2DM^32^. Similar results were found in current study.

Although this is the first study combining epidemiological research and meta-analysis to synthesize the prevalence, awareness, treatment and control of T2DM in Chinese rural population, some limitations should be noted. Firstly, estimations of the prevalence, awareness, treatment and control of T2DM were based on a cross-sectional study not prospective cohort design. A meta-analysis was conducted to confirm the results of this epidemiological research. The comparable results were found in both cross-sectional study and meta-analysis. Secondly, T2DM was defined according to fasting plasma glucose level or the use of insulin or anti-diabetic medications during the previous 2 weeks or the participants’ report of previous diagnosis of diabetes by a physician and not by oral glucose tolerance test (OGTT). Given that, the prevalence of T2DM may have been misestimated. Nonetheless, fasting plasma glucose is recommended in epidemiological field studies for its convenience, cost-effectiveness and acceptability among subjects^33^. Thirdly, the relationship between risk factors and T2DM may be affected by confounding factors. However, adjustment of a wide range of related risk factors allowed us to control for possible confounders in this study. Fourthly, the subjects of the present study had asymmetrical age structure, and were from the districts with higher economic level and living standard, which might lead to overestimate the prevalence, awareness, treatment and control of T2DM in Chinese rural areas. Thus, the participants of the RuralDiab study need to improve and perfect in the future study. Finally, the pooled age-standardized prevalence, awareness, treatment and control of T2DM in the meta-analysis were not given due to unavailable of the age-standardized prevalence, awareness, treatment and control in many of the selected studies. Although the present study has these limitations, the results based on the synthesizing epidemiological study and meta-analysis can represent the prevalence and influence factors of T2DM in Chinese rural areas.

In conclusion, the prevalence of T2DM is high, but the proportions of awareness, treatment and control are relatively low in China rural districts, which are slightly higher than the results of meta-analysis. Women have larger proportions of prevalence, awareness, treatment and control of T2DM than men. The prevalence of T2DM is significantly and positively associated with age, high risk alcohol drinking, being overweight or obese and having a family history of diabetes and negatively associated with higher education level, more vegetable and fruit intake and higher physical activity. Therefore, attention should be paid to healthy lifestyles to reduce the prevalence and improve awareness, treatment and control of T2DM in Chinese rural residents.

## Methods

### Study subjects

The subjects were from the Rural Diabetes, Obesity and Lifestyle (RuralDiab) study which was conducted in Yuzhou county in Xuchang city, Wuzhi county in Jiaozuo city, Houzhai village in Zhengzhou City and Xinan county in Luoyang city of Henan Province in China from July 2013 to August 2015. A total of 18817 participants completed the survey and physical examination from rural areas. To evaluate prevalence, awareness, treatment and control of T2DM of adults in rural areas, study subjects were excluded if they: (1) had type 1 diabetes mellitus (n = 8); (2) were aged <18 years or aged >74 years (n = 1311); and (3) did not have information about whether they were diagnosed diabetes: (n = 1085). Finally, 16413 subjects were included for the present study. This study was approved by the Zhengzhou University Ethics Committee. Informed consent was signed by all study participants or their agents. The present study was conducted according to the Declaration of Helsinki.

### Data collection and laboratory methods

Data were collected by face to face investigation. A standard questionnaire including information on general demographic characteristics (name, gender, living region, age, education level, marital status, occupation, family economic, medical services received, etc), life styles (intakes of vegetable, fruit, fat and protein intake, smoking, alcohol drinking, physical activity etc.), the personal history of diseases (hypertension, dyslipidemia, diabetes, coronary heart disease, stroke, etc), family history of diseases (hypertension, dyslipidemia, diabetes, coronary heart disease, stroke, etc) was completed by well-trained investigators.

Anthropometric measurements (height, weight, waist circumference, hip circumference and blood pressure) were performed. Height and body weight were measured with the subjects in light clothing and shoes off. Waist circumference at the level of 1.0 cm above the navel and hip circumference at the maximal level of the hip were measured with light clothing.

After at least 8 hours of overnight fasting, the fasting venous blood samples were collected for the measurement of plasma glucose. Blood samples were stored in −80 °C cryogenic refrigerator. FPG was measured by ROCHE Cobas C501 automatic biochemical analyzer with glucose oxidative method (GOD-PAP).

### Assessment of outcomes

T2DM was defined according to the American Diabetes Association (ADA) 2009^34^: FPG ≥7.0 mmol/L or self-reported use of insulin or anti-diabetic medications during the previous 2 weeks or participants reported he/she had previous diagnosis of diabetes by a physician. Awareness of those with T2DM was considered if they reported having a previous diagnosis of diabetes by physicians. The application of hypoglycemic medicine either insulin or oral pills during the previous 2 weeks was defined as treatment of those with T2DM. Individuals with T2DM were considered to have controlled plasma glucose if FPG ≤7.0mmol/L.

### Assessment of covariate factors

Education levels were classified as: ≤primary school (including those who never attended school, and those who attained primary school only); ≥junior school (including those who attained junior school or higher levels of education). In accordance with the smoking index (SI, SI = lifetime smoking intensity × duration of smoking) of the World Health Organization (WHO 1997), smoking was categorized into heavy smoking (SI ≥ 400), moderate smoking (200 ≤ SI < 400), light smoking (0 < SI < 200) and never smoking. Based on the daily intake amount of alcohol of China Public Union of Nutrition^35^ and WHO^36^ drinker was classified into never drinking, low risk drinking (0< ethanol/day ≤15g for women, 0< ethanol/day ≤25g for men), medium risk drinking (15< ethanol/day ≤40g for women, 25< ethanol/day l ≤ 60g for men), high risk drinking (ethanol/day >40g for women, ethanol/day >60g for men). More vegetable and fruit intake (adequate vegetable and fruit intake) was considered as a person who took more than 500g vegetable and fruit per day, and high fat diet was defined as a person who takes an average of more than 75g meat of livestock and poultry per day in line with the dietary guidelines for Chinese residents^35^. According to the international physical activity questionnaire (IPAQ 2001), physical activity was classified into low, moderate and high. BMI was divided into four levels: low weight, BMI <18.5 kg/m^2^; normal weight, 18.5≤ BMI <24 kg/m^2^; overweight, 24≤ BMI <28 kg/m^2^; and obese, BMI ≥28 kg/m^2^.

### Data extraction of meta-analysis

A meta-analysis was performed according to the guidelines of the Preferred Reporting Items for Systematic reviews and Meta-Analyses (PRISMA)^37^. Relevant studies in Web of Science, PubMed, CNKI (Chinese) and Wanfang (Chinese) databases surveyed from 1 January 2010 to 20 June 2016 (date of publication) were included by using the search terms ‘prevalence’, ‘awareness’, ‘treatment’, ‘control’, ‘type 2 diabetes mellitus or diabetes’, ‘China or Chinese’, ‘rural or countryside’ and these terms’ derivation and combinations. All pooled studies required to meet the following inclusion criteria: (1) the participants of studies were Chinese people (including Hong Kong, Macao and Taiwan); (2) the study had to report the number of total sample, prevalence, awareness, treatment and control of T2DM or diabetes of rural districts at the same time; and (3) based on population samples rather than volunteers. Exclusion criteria were: (1) reviews, editorials, commentaries, or reports etc; (2) duplication of studies, that is, repeating data that were already reported by other included articles ; and (3) studies focusing on special population, such as occupational crowds, health check-up population, ethnicity, or age groups. Two investigators independently extracted data information. Controversies were solved by discussion with necessity. For all selected studies, we extracted information regarding the first author, year of publication, study location, year of survey, age range, sampling method, diagnostic criteria, diagnosis method, total sample size (male/female), number of diabetes (male/female), prevalence (male/female), awareness (male/female), treatment (male/female), and control (male/female) of diabetes in rural districts.

### Statistical Analysis

All survey data were analyzed using SAS9.1 software package (SAS Institute, USA) and α = 0.05 was considered as significant difference. Descriptive statistics was used to summarize participants’ characteristics. Student t-test and chi-square test were applied to compare continuous and categorical variables, respectively. Odds ratios (*ORs*) and 95% confidence intervals (*CIs*) were used to explore the association between the socio-demographic factors and the prevalence, awareness, treatment and control of T2DM. All selected characteristics were included in multivariable logistic regression models. Direct standardized methods using the Population Census 2010 were utilized to estimate age-standardized prevalence, awareness, treatment and control of T2DM.

The meta-analysis was performed using the Stata Software Package, V11.0 (StataCorp, College Station, Texas, USA). Pooled estimates of diabetic prevalence, awareness rate, treatment rate, and control rate and their corresponding 95% confidence intervals (*CIs*) were calculated based on the random effect model. Statistical heterogeneity was evaluated by the *I*^*2*^statistic (low, *I*^*2*^ < 25%; moderate, 25–50%; high, *I*^*2*^ > 50%)^38^. To explore potential publication bias, the Begg’s and Egger’s tests were conducted.

## Additional Information

**How to cite this article**: Liu, X. *et al*. Prevalence, awareness, treatment, control of type 2 diabetes mellitus and risk factors in Chinese rural population: the RuralDiab study. *Sci. Rep*. **6**, 31426; doi: 10.1038/srep31426 (2016).

## Supplementary Material

Supplementary Information

## Figures and Tables

**Table 1:** Demographic characteristics.

Variable	T2DM group (N = 2000)	Control group (N = 14413)	*χ*^*2*^*/t*	*P*
Age (years), mean ± SD	59.13 ± 8.73	53.95 ± 11.50	286.52	<0.001
Men, n (%)	702 (35.10)	5273 (36.59)	1.67	0.196
Marital status, n (%)			4.42	0.036
Married/cohabiting	1780 (89.40)	13048 (90.86)		
Widowed/single/divorced/separation	211 (10.60)	1312 (9.14)		
Education, n (%)			100.64	<0.001
≤Primary school	1110 (55.53)	6280 (43.61)		
≥Junior school	889 (44.47)	8119 (56.39)		
Smoking, n (%)			16.65	0.001
Never	1541 (79.84)	10653 (76.46)		
Light	58 (3.01)	605 (4.34)		
Moderate	58 (3.01)	587 (4.21)		
Heavy	273 (14.15)	2087 (14.98)		
Drinking, n (%)			18.58	<0.001
Never	1715 (85.75)	11994 (83.22)		
Low risk	170 (8.50)	1670 (11.59)		
Medium risk	57 (2.85)	412 (2.86)		
High risk	58 (2.90)	337 (2.34)		
More vegetable and fruit intake, n (%)	358 (17.92)	3203 (22.22)	19.15	<0.001
High fat diet, n (%)	262 (13.10)	2248 (15.60)	8.45	0.004
Physical activity, n (%)			155.84	<0.001
Low	998 (49.90)	5276 (36.61)		
Moderate	370 (18.50)	2622 (18.19)		
High	632 (31.60)	6514 (45.20)		
Family history, n (%)	265 (13.25)	1018 (7.06)	93.30	<0.001
BMI (kg/m^2^), mean ± SD	26.39 ± 3.73	25.07 ± 3.55	4.49	0.034

**Table 2:** Comparison of prevalence, awareness, treatment and control of T2DM between various factors (N = 16413).

Variable	Prevalence (n = 2000)	T2DM (n = 2000)		
		Awareness (n = 1340)	Treatment (n = 1247)	Control (n = 444)
Age (years), n (%)				
18~	11 (1.31)	2 (18.18)	1 (9.09)	1 (9.09)
35~	120 (5.34)	55 (45.80)	47 (39.17)	11 (9.17)
45~	441 (9.43)	267 (60.54)	243 (55.10)	70 (15.87)
55~	834 (16.39)	579 (69.42)	548 (65.71)	196 (23.50)
65~74	594 (16.70)	437 (73.57)	408 (68.69)	166 (27.95)
*P*	<0.001	<0.001	<0.001	<0.001
*P*_*trend*_	<0.001	<0.001	<0.001	<0.001
Gender, n (%)				
Women	1298 (12.44)	907 (69.88)	848 (65.33)	316 (24.35)
Men	702 (11.75)	433 (61.68)	399 (56.84)	128 (18.23)
*P*	0.196	<0.001	<0.001	0.002
Marital status, n (%)				
Married/cohabiting	1780 (12.00)	1194 (67.08)	1114 (62.58)	390 (21.91)
Widowed/single/divorced/separation	211 (13.85)	138 (65.40)	126 (59.72)	54 (25.59)
*P*	0.036	0.625	0.416	0.224
Education, n (%)				
≤Primary school	1110 (15.02)	783 (70.54)	730 (65.77)	266 (23.96)
≥Junior school	889 (9.87)	556 (62.54)	516 (58.04)	178 (20.02)
*P*	<0.001	<0.001	<0.001	0.035
Smoking, n (%)				
Never	1541 (12.64)	1071 (69.50)	1004 (65.15)	366 (23.75)
Light	58 (8.75)	35 (60.34)	32 (55.17)	11 (18.97)
Moderate	58 (8.99)	32 (55.17)	28 (48.28)	7 (12.07)
Heavy	273 (11.57)	160 (58.61)	144 (52.75)	47 (17.22)
*P*	0.001	<0.001	<0.001	0.020
*P*_trend_	0.020	<0.001	<0.001	0.005
Drinking, n (%)				
Never	1715 (12.51)	1189 (69.33)	1112 (64.84)	397 (23.15)
Low risk	170 (9.24)	90 (52.94)	80 (47.06)	27 (15.88)
Medium risk	57 (12.15)	31 (54.39)	27 (47.37)	8 (14.04)
High risk	58 (14.68)	30 (51.72)	28 (48.28)	12 (20.69)
*P*	<0.001	<0.001	<0.001	0.069
*P*_trend_	0.626	<0.001	<0.001	0.101
More vegetable and fruit intake, n (%)	358 (10.05)	238 (66.48)	220 (61.45)	70 (19.55)
*P*	<0.001	0.829	0.711	0.180
High fat diet, n (%)	262 (10.44)	156 (59.54)	143 (54.58)	38 (14.50)
*P*	0.004	0.006	0.005	0.001
Physical activity, n (%)				
Low	998 (15.91)	701 (70.24)	658 (65.93)	239 (23.95)
Moderate	370 (12.37)	246 (66.49)	231 (62.43)	80 (21.62)
High	632 (8.84)	393 (62.18)	358 (56.65)	125 (19.78)
*P*	<0.001	0.003	0.003	0.137
*P*_trend_	<0.001	0.001	0.001	0.046
Family history, n (%)	265 (20.65)	211 (79.62)	190 (71.70)	58 (21.89)
*P*	<0.001	<0.001	0.001	0.895
Body mass index, n (%)				
Underweight	19 (6.93)	15 (78.95)	14 (73.68)	2 (10.53)
Normal	511 (8.55)	350 (68.49)	322 (63.01)	111 (21.72)
Overweight	825 (12.40)	564 (68.36)	528 (64.00)	196 (23.76)
Obese	612 (17.92)	381 (62.25)	354 (57.84)	129 (21.08)
*P*	<0.001	0.037	0.066	0.368
*P*_*trend*_	<0.001	0.012	0.037	0.998

**Table 3:** Results of multivariate logistic regression for prevalence, awareness, treatment and control of T2DM.

Variable	Prevalence *OR95%CI*	T2DM		
		Awareness *OR95%CI*	Treatment *OR95%CI*	Control *OR95%CI*
Age (years)				
18~	1.00	1.00	1.00	1.00
35~	3.94 (2.10–7.40)	3.07 (0.61–15.50)	5.68 (0.69–47.09)	0.81 (0.09–7.16)
45~	7.75 (4.21–14.27)	5.89 (1.20–28.84)	11.00 (1.36–89.03)	1.44 (0.16–11.75)
55~	15.89 (8.64–29.21)	8.99 (1.83–44.15)	17.38 (2.15–140.89)	2.32 (0.29–18.86)
65~74	15.93 (8.62–29.46)	11.35 (2.29–56.29)	20.42 (2.50–166.63)	3.12 (0.38–25.55)
*P*_trend_	<0.001	<0.001	<0.001	<0.001
Gender				
Women	1.00	1.00	1.00	1.00
Men	1.11 (0.96–1.29)	0.97 (0.72–1.30)	1.03 (0.77–1.37)	0.81 (0.58–1.13)
Marital status				
Married/cohabiting	1.00	1.00	1.00	1.00
Widowed/single/divorced/separation	0.94 (0.80–1.11)	0.78 (0.57–1.07)	0.74 (0.55–1.01)	1.05 (0.75–1.48)
Education				
≤Primary school	1.00	1.00	1.00	1.00
≥Junior school	0.89 (0.79–0.99)	0.93 (0.74–1.17)	0.98 (0.79–1.23)	1.18 (0.91–1.52)
Smoking				
Never	1.00	1.00	1.00	1.00
Light	0.88 (0.65–1.20)	1.09 (0.59–2.01)	1.01 (0.56–1.85)	1.08 (0.51–2.27)
Moderate	0.86 (0.63–1.18)	0.89 (0.48–1.65)	0.84 (0.45–1.55)	0.61 (0.26–1.45)
Heavy	0.85 (0.71–1.03)	0.86 (0.59–1.24)	0.79 (0.55–1.13)	0.86 (0.55–1.35)
*P*_trend_	<0.001	<0.001	<0.001	0.005
Drinking				
Low risk	1.00	1.00	1.00	1.00
Never	1.18 (0.97–1.43)	1.74 (1.19–2.54)	1.83 (1.26–2.66)	1.27 (0.77–2.09)
Medium risk	1.15 (0.82–1.60)	1.28 (0.67–2.44)	1.20 (0.63–2.27)	0.94 (0.37–2.34)
High risk	1.62 (1.16–2.27)	1.13 (0.60–2.13)	1.27 (0.68–2.40)	1.93 (0.87–4.28)
*P*_trend_	<0.001	0.920	0.913	0.626
More vegetable and fruit intake	0.86 (0.76–0.98)	1.08 (0.84–1.41)	1.08 (0.84–1.40)	0.91 (0.67–1.22)
High fat diet	1.04 (0.89–1.20)	0.90 (0.66–1.22)	0.96 (0.71–1.28)	0.70 (0.48–1.03)
Physical activity				
Low	1.00	1.00	1.00	1.00
Moderate	0.86 (0.75–0.98)	0.88 (0.68–1.16)	0.91 (0.70–1.18)	0.95 (0.71–1.28)
High	0.61 (0.54–0.68)	0.76 (0.60–0.95)	0.73 (0.59–0.91)	0.91 (0.71–1.18)
*P*_trend_	<0.001	0.001	<0.001	0.046
Family history	3.07 (2.61–3.60)	2.93 (2.08–4.11)	2.22 (1.63–3.02)	1.22 (0.88–1.70)
Body mass index				
Normal	1.00	1.00	1.00	1.00
Underweight	0.72 (0.44–1.16)	1.68 (0.54–5.20)	1.57 (0.55–4.49)	0.39 (0.09–1.73)
Overweight	1.46 (1.29–1.64)	1.06 (0.83–1.36)	1.10 (0.86–1.40)	1.22 (0.93–1.60)
Obese	2.31 (2.02–2.63)	0.87 (0.67–1.13)	0.71 (0.71–1.18)	1.07 (0.80–1.44)
*P*_trend_	<0.001	0.044	0.120	0.981
