# Supplementary material for: Prevalence, awareness, treatment, control of type 2 diabetes mellitus and risk factors in Chinese rural population: the RuralDiab study
Source: Sci Rep. 2016 Aug 11;6:31426. doi: 10.1038/srep31426 (PMC4980764; doi:10.1038/srep31426)
Supplement: Supplementary Information [file srep31426-s1.pdf]

Title

Prevalence, awareness, treatment, control of type 2 diabetes mellitus and risk factors in Chinese rural population: the RuralDiab study

Short title:

Type 2 diabetes mellitus in rural China

Authors:

Xiaotian Liu[1], Yuqian Li[2], Lin-lin Li[1], Luning Zhang[1], Yongcheng Ren[1], Hao Zhou[1], Lingling Cui[3], Zhenxing Mao[1], Dongsheng Hu[1,4], Chongjian Wang[1]

Authors affiliations:

[1] Department of Epidemiology and Biostatistics, College of Public Health, Zhengzhou University, Zhengzhou, Henan, *PR* China.

[2] Department of Clinical Pharmacology, School of Pharmaceutical Science, Zhengzhou University, Zhengzhou, Henan, *PR* China.

[3] Department of Nutrition and Food Hygiene, College of Public Health, Zhengzhou University, Zhengzhou, Henan, *PR* China.

[4] Department of Prevention Medicine, Shenzhen University School of Medicine, Shenzhen, *PR* China.

*** Correspondence author**

Dr. Chongjian Wang

Department of Epidemiology and Biostatistics

College of Public Health, Zhengzhou University

100 Kexue Avenue, Zhengzhou, 450001, Henan, *PR* China

Phone: +86 371 67781452;

Fax: +86 371 67781868

E-mail: tjwcj2005@126.com

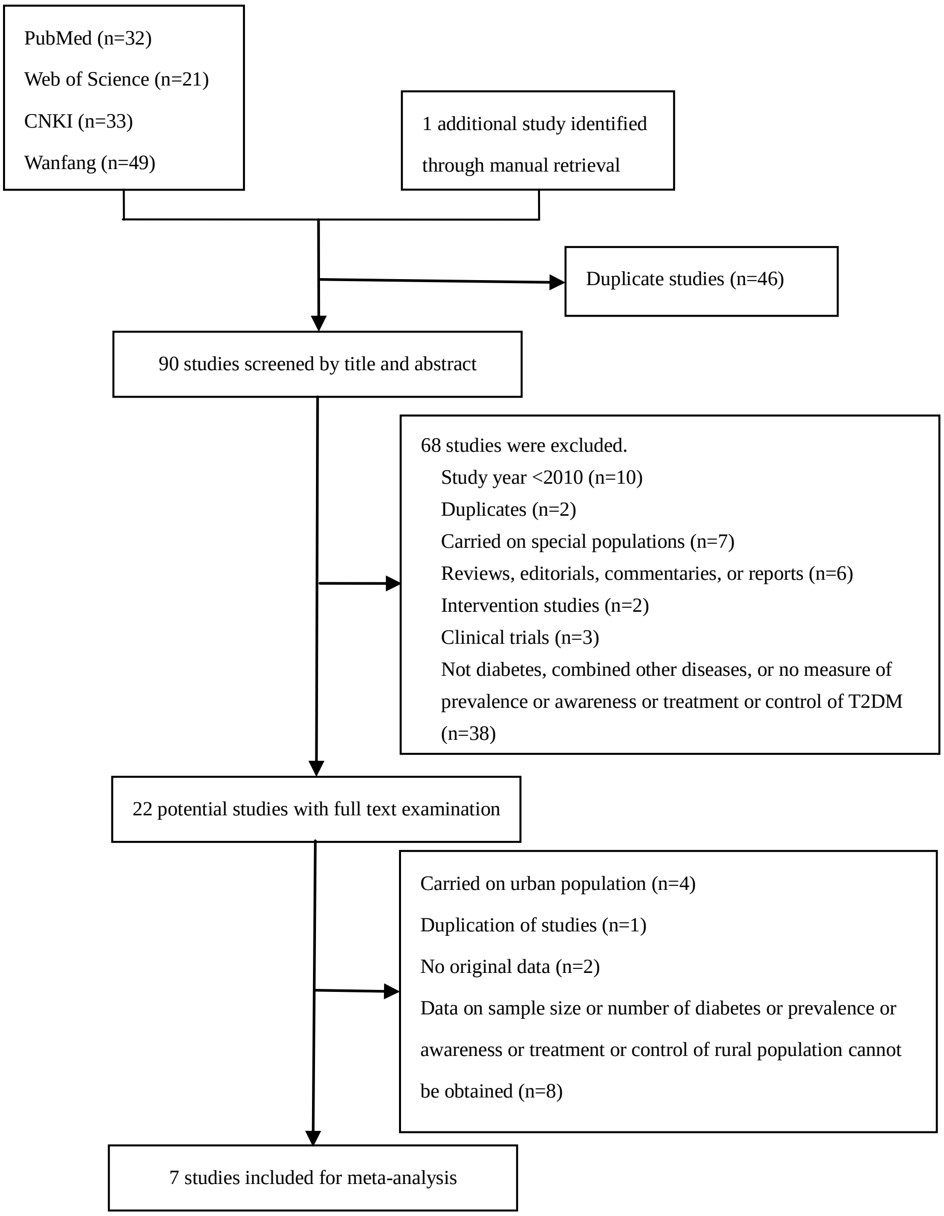

Supplementary Figure S1 Flow diagram of included studies for meta analysis

First author & published year	Study location	Survey data	Age range	Location	Sampling method	Diagnostic criteria	Diagnosis method	Total sampling size (male/female)	Total case size (male/female)	Prevalence (male/female),%	Awareness (male/female),%	Treatment (male/female),%	Control (male/female),%
Wang 2016[19]	Jilin	2012	18-79	Rural	Multistage stratified cluster	FBG≥7.0mmol/L/ anti-diabetic medications	FPG	9600 (4884/4716)	687 (339/348)	7.2 (6.9/7.4)	69.0 (60.2/77.6)	60.8 (51.6/69.8)	**25.9 (21.2/30.5)**
Feng 2015[20]	Guizhou	Oct.~Dec.2012	20~80	Rural	Cluster sampling	ADA2014	FPG	1060 (440/620)	40 (21/19)	3.77 (4.77/3.06)	30.00	25.00	7.50
Wang 2014[21]	Jilin	2013-2014	35~	Rural	Cluster sampling	FBG≥7.0mmol/L/ anti-diabetic medications/ previous diagnosis of diabetes	FPG	2600 (1179/1421)	190 (85/105)	7.3 (7.2/7.4)	54.7 (44.7/62.9)	48.9 (38.8/57.1)	14.7 (10.6/18.1)
Wang 2014[22]	Jilin	Jul.~Aug.2012	18~79	Rural/urban	Multistage stratified random cluster sampling	China guideline for type 2 diabetes(2007)	FPG	8745	923 (412/511)	10.6	65.8 (59.5/76.5)	52.7 (50.2/63.8)	23.9 (18.9/28.0)
Xu 2013[15]	31 province	2010	18~	Rural/urban	Multistage random cluster sampling	ADA 2010	FPG/OGTT/ HbA_{1c}	59730	6521	10.9	27.3	23.8	8.7
Wang 2012[23]	Suzhou	2010	20~80	Rural/urban	Multistage random cluster sampling	WHO1999	FPG	23349	1705	7.30	69.03	51.96	37.89
Zhang 2011[24]	Jiangsu	2010	35~	Rural	Multistage sampling	WHO1999	FPG	4634 (1883/2751)	194 (78/116)	4.19 (4.14/4.22)	80.41 (79.49/81.03)	69.07 (73.07/66.38)	27.32 (28.20/26.72)

WHO: World Health Organization; ADA: American Diabetes Association; FPG: fasting plasma glucose; OGTT: oral glucose tolerance test; HbA_{1c}: glycated hemoglobin A_{1c}.

Supplementary Table S1 Characteristics of selected studies
